# Epidemiological study on animal bite cases referred to Haji Daii health Center in Kermanshah province, Iran during 2013–2017

**DOI:** 10.1186/s12889-020-08556-1

**Published:** 2020-03-30

**Authors:** Maryam Janatolmakan, Mojtaba Delpak, Alireza Abdi, Sabah Mohamadi, Bahare Andayeshgar, Alireza Khatony

**Affiliations:** 1 Clinical Research Development Center, Imam Reza Hospital, Kermanshah University of Medical Sciences, Kermanshah, Iran; 2 Student Research Committee, Kermanshah University of Medical Sciences, Kermanshah, Iran; 3 Infectious Diseases Research Center, Kermanshah University of Medical Sciences, Kermanshah, Iran

**Keywords:** Epidemiology, Animals, Bite, Iran

## Abstract

**Background:**

Over thousands of animal bite cases are reported annually worldwide and in Iran placing a large financial burden on the health and economy. The aim of this study was to evaluate the epidemiology of animal bite cases in Kermanshah, Iran through 2013–2017.

**Methods:**

In this cross-sectional study, 5618 animal bite cases in Kermanshah from 2013 to 2017 were studied. Data were analyzed using descriptive and inferential statistics.

**Results:**

In the study period, 5618 animal bite cases were found. The prevalence of animal bites was estimated between 42.55–45.66 per100000 populations during 2013–2017. An increasing significant trend was found for prevalence of animal bites (Average annual percent change [AAPC] + 4.9, *P*-trend< 0.001) over a 5-years’ time period. The mean age of the subjects was 32.7 ± 18.3 years. Of the studied subjects 76.3% were male, and 34% had non-governmental jobs. Dogs were found as the cause of animal bites in 72% of the cases. Of the studied cases, 82% had received rabies vaccination for three times.

**Conclusion:**

The results showed an increasing significant trend for animal bites in Kermanshah. Development of interventional programs, such as limiting stray dogs, vaccination of dogs and raising public awareness are essential.

## Background

Animal bites are one of the leading causes of death worldwide [1, 2]. Animal saliva is composed of a wide range of pathogenic infectious bacteria that can transmit several lethal infections, such as rabies to humans [2, 3]. According to the Centers for Disease Control and Prevention, around 4.5 million people worldwide are bitten by animals every year and often postexposure prophylaxis is needed [2]. Epidemiological evidences suggest that more than 2.5 billion people are at risk of rabies, according to the World Health Organization [4, 5]. Each year, about 10 million people receive post exposure rabies vaccination. Around 50–60,000 rabies-related deaths are reported annually worldwide, of which 31–32,000 are occurred in the Asia and Africa [2, 3, 6–8]. The prevalence of animal bites in Iran has been reported between 98 and 450 people per 100,000 populations over the years 2008–2014 [9–13]. Several million dollars are spent annually on preventing rabies in Iran and no other contagious disease can be found in Iran that costs as much as rabies [14–16]. The growing trend of stray dogs populations and also increasing numbers of animal bite cases and rabies in many provinces of Iran, indicate the importance of paying more attention to their management and investigations on their different aspects [12, 17]. Accurate information regarding the epidemiological status of the disease is needed for effective prevention programs [12, 18]. Given the lack of epidemiological information on animal bite cases in Kermanshah, Iran, a cross-sectional study was conducted to estimate the prevalence of animal bite cases and identify factors associated with higher prevalence of animal bites in Kermanshah, Iran through 2013–2017.

In this study, we sought to answer questions about the demographic information of the bite victims, locations of animal bite, types of animal bite, the season of bite occurrence, the situation of the victims during the animal attack, and the prevalence of animal bites during 2013–2017.

## Methods

### Study sites

**Fig. 1:**
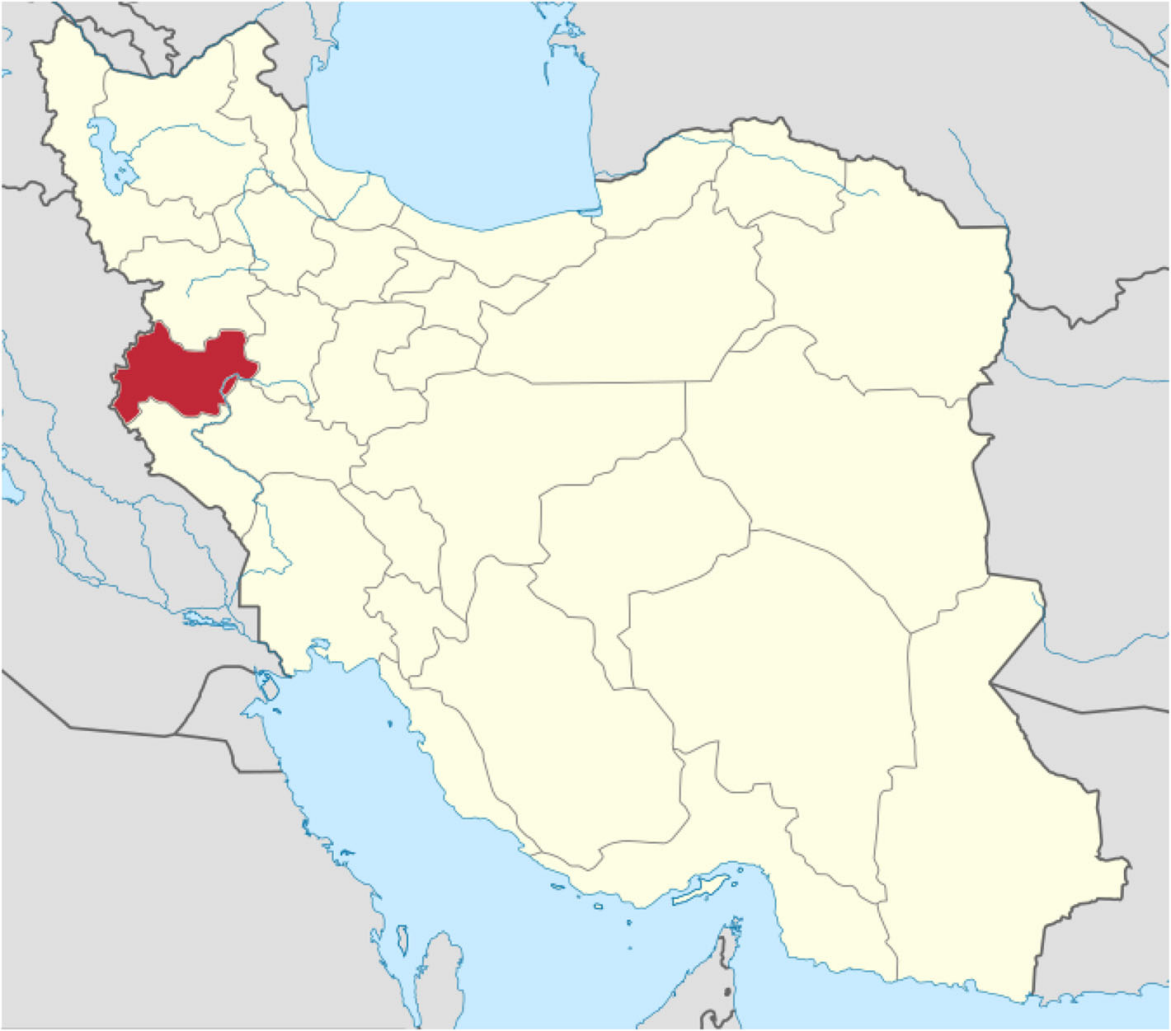
Map of Iran; Kermanshah province is marked in red. Source: https://commons.wikimedia.org/wiki/File:Locator_map_Iran_Kermanshah_Province.png

This study was conducted in Kermanshah province, Iran. Kermanshah province is located in the west of Iran and covers an area of 24.640 km^2^. It is the seventeenth province of Iran in terms of size (Fig. 1). Kermanshah is the ninth most populous province in Iran, with a population of 1,900,000 people (according to the 2016 census) [12]. It is one of the tribal areas in Iran including 14 counties and 84 villages. The counties include Kermanshah, Dalahu, Gilan-e Gharb, Harsin, Eslamabad-e Gharb, Javanrud, Kangavar, Paveh, Qasr-e Shirin, Ravansar; Sahneh, Sarpol-e Zahab, Salas-e Babajani, and Sonqor [19]. Haji Daii Clinic located in Kermanshah is the main site for animal bite registration in Kermanshah province. For all victims, a case is being prepared at this location and necessary medical treatment such as rabies vaccination is provided.

### Study population

The number of animal bite cases in Kermanshah through 2013–2017 was 5618. Inclusion criteria were whom referred to one of the health clinics and had a history record. The records with incomplete information were excluded if the patient did not answer to fulfill the form.

### Study design/data management

**Fig. 2:**
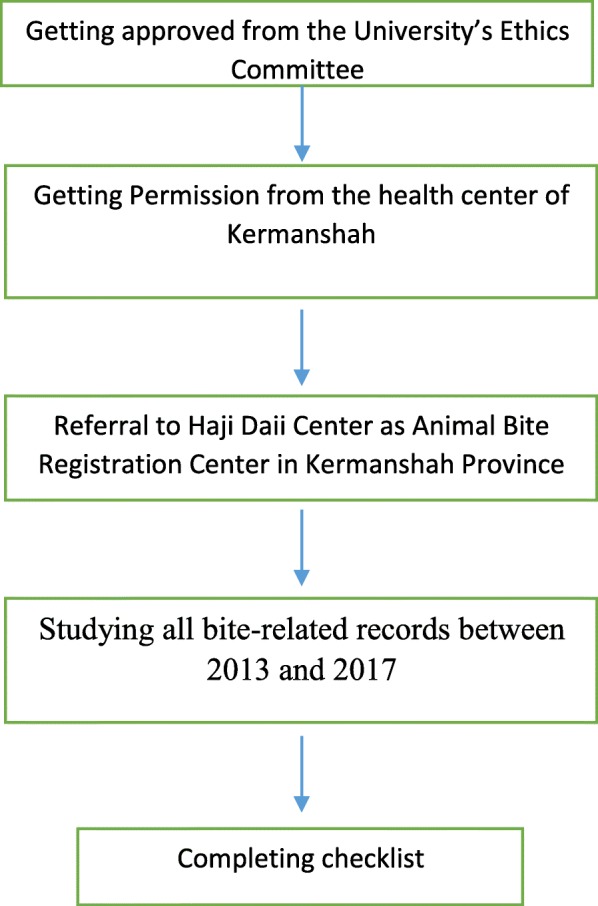
Flow diagram of study

This cross-sectional study was carried out in Kermanshah, Iran, and was based on the STROBE guideline***.*** The research tool was a researcher-made checklist containing 11 questions assessing age, sex, occupation, place of residence, where animal bites happened, affected site, type of animal, frequency of rabies vaccination, the year, the season and the victim situation at the time of bite. After obtaining approval from the University Ethics Committee, the researcher referred to the Haji Dae Clinic for studying all bite-related records between 2013 and 2017 and included the essential information in the checklist (Fig. 2). Chi-square test was used to determine the relationship different between nominal and categorical variables in terms of number of bites. The prevalence of animal bites in each year was calculated as the number of cases divided by the population of that year multiplied by 100,000. It is notable the population number was estimated based on the data of Statistical center of Iran (https://www.amar.org.ir/english) and their census results and growth rate. The trend was tested using the Cochran-Armitage test in Stata software.

### Ethical consideration

The study was approved by the Ethics Committee of Kermanshah University of Medical Sciences with the code: KUMS.REC.1397.170*.* Permission was also obtained from the health center of Kermanshah.

## Results

The total of 5618 animal bite cases was recorded through 2013–2017 and there were 14 incomplete records. The prevalence of animal bite was obtained 42.55–45.66 per 100,000 populations, during the studied period, and its average was 44.1. The mean age of subjects was 32.7 ± 18.3 years with the age range of 27–39 years in most subjects (*n* = 1467, 26.1%) (Table 1). Except for the age group of 1–9 year, an increasing trend of animal bites prevalence was found for other age groups during the study period, which was significant for 30–39 and 40–49 age groups (Table 2). In the age range of 20–29 year, the prevalence of animal bite was 1.40 times higher than that of 1–19 year (Table 3). Of those who had been bitten 4277 subjects (76.3%) were male. The prevalence of animal bites in both genders (Average annual percent change [AAPC] + 4.7 for male and + 5.32 for female) increased significantly over the study time period (Table 2). The prevalence of animal bites in men was 3.15 times higher than in women (Table 3).

**Table 1:** Prevalence of animal bites based on the demographic variables

Variable		Number (%)	Test result
Occupation	Employee	533 (9.5)	X^2^ = 564 *P* < 0.001
	Self-employed^*^	1904 (34.0)	
	Farmer	347 (6.2)	
	Student	1204 (21.5)	
	Unemployed	178 (3.2)	
	Ranchman	87 (1.5)	
	Housewife	812 (14.5)	
	Child	310 (5.5)	
	Retired	161 (2.9)	
	Soldier	68 (1.2)	
location of wound	Upper limbs^*^	2776 (49.5)	X^2^ = 234 *P* < 0.001
	Lower limbs	2666 (47.6)	
	Both limbs	162 (3.0)	
Species	Dog^*^	4032 (72.0)	X^2^ = 481 *p* < 0.001
	Cat	1194 (21.3)	
	Livestock^a^	41 (0.7)	
	Others^b^	335 (6.0)	
Number of vaccination against rabies	Three	4594 (82.0)	X^2^ = 229 *P* < 0.001
	Five	1009 (18.0)	
Frequency of animal bite for each Year	2013	1017 (18.1)	X^2^ = 25.2 *P* < 0.001
	2014	1072 (19.1)	
	2015	1117 (19.9)	
	2016	1161 (20.7)	
	2017^*^	1237 (22.1)	
Season	Spring^*^	1600 (28.5)	X^2^ = 73.1 *P* < 0.001
	Summer	1495 (26.7)	
	Fall	1329 (23.7)	
	winter	1180 (21.1)	
Situation	Sudden animal attack^*^	3302 (58.9)	X^2^ = 778 *P* < 0.001
	Animal stimulation by humans	1021 (18.2)	
	When feeding the animal	412 (7.3)	
	When playing with animals	564 (10.1)	
	When taking care of animal	256 (4.6)	
	During human rest	48 (0.8)	
Location of animal attack	Rural	1365 (24.0)	X^2^ = 312 *P* < 0.001
	Urban^*^	4239 (76.0)	

^*^Based on the post hoc test, it was significantly higher than the others

^a^Including donkey, horse, cow and sheep

^b^Including monkeys, mice, hamster, and other wild animals

**Table 2:** Trends of animal bites prevalence (per 100,000) by sex, location of animal attack, and age, during 2013–2017

Variables		Number (%)	Years of study					AAPC^a^	*P*-value
			2013	2014	2015	2016	2017		
Population			2,227,090	2,382,986	2,549,795	2,728,281	2,819,261		
Sex	Male	4277 (76.3)	79.1	85.6	86.8	87.8	95.0	5.0	0.005
	Female	1324 (24.0)	25.1	24.2	27.5	30.3	31.0	5.3	0.013
Location of animal attack	Rural	1365 (24.0)	52.3	58.5	59.0	60.3	63.7	4.6	0.002
	Urban	4239 (76.0)	53.1	46.6	54.3	57.3	63.1	5.0	0.010
Age groups (year)	1–9	599 (11.0)	38.4	43.1	41.6	45.5	37.3	−0.7	0.513
	10–19	772 (14.0)	55.5	52.5	53.2	54.0	68.0	5.1	0.115
	20–29	1347 (24.0)	62.2	62.4	70.0	70.4	77.0	5.5	0.063
	30–39	1050 (19.0)	50.3	53.1	60.4	63.0	64.6	6.4	0.045
	40–49	706 (13.0)	49.1	46.8	54.1	62.7	61.6	5.9	0.045
	50–59	576 (10.2)	57.4	67.6	62.3	56.1	68.3	4.5	0.434
	> 60	568 (10.1)	50.1	66.5	56.4	59.0	66.9	7.5	0.183
Total		5618	52.4	55.2	57.5	59.4	63.4	4.9	< 0.001

^a^Average annual percent change

**Table 3:** Prevalence odds ratio for demographic variables

Variable		Odds ratio	95% confidence interval
Sex	Female	Reference	
	Male	3.15	2.96, 3.35
Location of animal attack	Rural	Reference	
	Urban	1.07	1.01,1.14
Age groups (year)	1–19	Reference	
	20–29	1.40	1.30,1.51
	30–39	1.20	1.11,1.30
	40–49	1.13	1.03,1.24
	> 50	1.26	1.16,1.36
Year of study	2013	Reference	
	2014	1.05	0.96, 1.14
	2015	1.09	1.01, 1.19
	2016	1.13	1.04,1.23
	2017	1.21	1.11, 1.31

In terms of occupation, the highest and the lowest rates of animal bite were recorded for those with non-governmental jobs (*n* = 1904, 34.1%) and soldiers (*n* = 68, 1.2%) respectively. According to the results of the post-hoc test, the prevalence of animal bite in victims with non-governmental jobs was significantly higher than the others (*p* < 0.001). The majority of victims were bitten in the urban areas (*n* = 4239, 76.0%) (Table 1). The prevalence of animal bites in urban/rural residency increased significantly over the study time period (AAPC for urban + 5.03 and for rural + 4.6)(Table 2). The prevalence of animal bites in the urban population was 1.07 times higher than in rural areas (Table 3). Of the studied cases, 4032 cases (72%) and 1194 cases (21.3%) were bitten by dogs and cats, respectively. The prevalence of animal bites from dogs was significantly higher than others according to the post-hoc test (*p* < 0.001). In 3304 cases (58.9%) a sudden animal attack were reported which was significantly higher than other situations according to the post-hoc test (*p* < 0.001). In 1021 cases (18.2%) the animals was stimulated by humans. In our study, 49.5% of bites (2776 cases), had occurred in the upper limbs. There was a significant difference between the prevalence of upper and lower extremity injuries according to the post hoc test. Most victims (*n* = 4594, 82%) had vaccinated with rabies vaccine for three doses times (Table 1). The highest and lowest frequency of animal bite cases were recorded in 2017 (*n* = 1237, 22%) and 2013 (*n* = 1017, 18.1%), respectively. An increasing significant trend was found for incidence of animal bites over a 5-year time period (AAPC + 4.9, *P*-trend< 0.001) (Table 2). The prevalence of animal bite in the year 2017 was 1.21 times higher than in the year 2013 (Table 3).

The highest (*n* = 1600, 28.5%) and the lowest (*n* = 1180, 21%) number of animal bite cases were reported in spring and winter, respectively. According to the results of post-hoc test, the prevalence of animal bite in spring was significantly higher than in autumn and winter (*p* < 0.001).

## Discussion

Animal bite is one of the major causes of mortality and also a major health problem worldwide [20]. The aim of this study was to investigate the epidemiology of animal bite cases in Kermanshah province during 2013–2017. The results showed that in the studied period, 5618 people had been bitten and an increasing significant trend was found for prevalence of animal bites over a 5-year time period. The mean prevalence of animal bites was estimated 44.1 per 100,000 populations during this time. In 2017, the prevalence of animal bites was 1.21 times higher than in 2013. In Holzer et al. study (2019) in the United States, the prevalence of animal bite cases between 2010 and 2014 was 0.25 and 0.19%, respectively [13]. Venkatesan et al. (2014) study in India indicated the prevalence of 81.8% per 1000 population for animal bite [21]. The high incidence of animal bites indicates the need for serious consideration to this issue. In this regard, health authorities should take the necessary measures to limit stray dogs, vaccine dogs, and develop training programs to make people inform about the complications of animal bites and how to prevent them. The animal bite cases have been increasing in Kermanshah through 2013–2017, which is consistent with the results of Frey et al. in Chad [2]. The results of a study by Cuc et al. (2018) in Haiti reported 690 animal bite cases within 6 months [22]. The results of Zohrevandi et al. (2012) study in Gilan, Iran showed that 1014 cases of animal bite were recorded in 2012 [23]. According to the Charkazi et al. (2013) study in Golestan, Iran, through 1998–2009 13,142 animal bites were reported [24]. In Kermanshah province, livestock farming is common and there is a high number of stray animals such as dogs and cats, so the possibility of animal bites is high. On the other hand, the growing number of animal bites shows that despite significant advances in health carein Kermanshah province, it has not yet been effective.

In our study, the prevalence of animal bites in both genders increased significantly over the study time period, which was 3.15 times higher in males than females. This finding is consistent with the results of other studies [9, 11, 25, 26]. The high prevalence of animal bite in male subjects can be associated with the large number of tribes living in Kermanshah. Accordingly, men are more likely to be bitten by animals as they are more active in outdoor activities.

In the current study, the prevalence of animal bites in urban/rural residency increased significantly over the study time period, which was 1.07 higher in urban areas than rural areas. This finding is consistent with the results of the Patel et al. (2017) and Riahi et al. (2012) studies in India and Iran, respectively [27, 28]. However, in some studies, the higher prevalence of animal bite cases were observed in rural areas [11, 23, 29]. In our opinion, the higher prevalence of animal bites in urban areas compared to rural areas may be due to the fact that urban dwellers do not know how to treat animals.

In the present study, except for the age group of 1–19 year old, an increasing trend of animal bites prevalence was found for other age groups during the study period, which was significant for 30–39 and 40–49 age groups. The prevalence of animal bites in the age groups of 30–39 and 40–49 was 1.2 and 1.13 times higher than that in the age group of 1–19, respectively. In most studies, the age group of younger than 40 years is the most common group that gets bitten by animals [3, 6, 11, 27, 30–32]. Those who are in the age range of 30–49 years are more active and adventurous, so animals can be stimulated to attack. They are also more present in the community, which can make them more vulnerable to animal bites.

In our study, the upper extremities were more involved than the lower extremities, and this difference was significant by the post hoc test. This finding is in line with the results of Shuzhen et al. (2018) study in Shenzhen and Shantou cities in China and also Zohrevandi et al. (2012) in Guilan, Northern Iran [23, 33]. However, some studies reported lower extremities as the most common affected site [8, 26, 27, 30, 34]. During an animal attack, both upper and lower extremities may be affected, which may be related to the position of the victim during the animal attack.

Based on the results, dogs were the most common cause for animal bite, which is similar to other studies [3, 11, 23, 27, 31, 34, 35]. The high prevalence of animal bite by dogs can be associated with the large number of tribes living in Kermanshah as well as the presence of stray dogs through the city. A sudden animal attack was the most common type of exposure to animal bite. Its high incidence can indicate that most cases were occurred without any special reason or stimulation.

Most cases had vaccinated with rabies vaccine for three times. In other studies, vaccination and human Rabies Immunoglobulin (HRIG) vaccination were reported as post-exposure measures [3, 9, 22, 27, 36, 37]. Vaccination and administration of HRIG, along with basic measures, such as washing with water and soap, can prevent the risk of animal bites, including the possibility of rabies [21, 38]. To schedule a rabies vaccination, 0.5 cc of the vaccine is inoculated into the deltoid muscle for five times on the first, third, seventh, fourteenth, and twenty-eighth days post exposure [39].

In the current study, the most common animal bite season was spring, which is consistent with some other studies [23, 40, 41]. The high prevalence of animal bites in the spring can be due to the presence of more people in recreational areas and outdoors, which makes them more likely to face animals.

In terms of occupation, the most victims had non-governmental jobs, however in some studies, students and university students were shown as the most common victims [11, 27]. The high incidence of animal bites in people with non-governmental jobs may be due to their working conditions, since they actually spend more time outdoors and are more likely to be exposed by animals.

Incomplete information recorded in victims’ documents was one of the main limitations of this study. Accordingly, they were contacted to answer incomplete information as far as possible. Other possible limitation was related to the possibility of inaccurate transferring the records data into the checklist, herewith the researcher tried to recheck all the information at least twice. The differing health status of different countries as well as laws related to animals affect the generalizability of our results.

## Conclusion

The prevalence animal bites in the studied period was estimated between 42.55–45.66 per100000 populations. The highest and lowest number of animal bites had recorded in 2017 and 2013, respectively, indicating it upward trend. An increasing significant trend was found for prevalence of animal bites over a 5-years’ time period. The highest prevalence of animal bites was observed in those with non-governmental jobs and also in male subjects, youth and urban residents. Spring was the most common season for animal bites. Similar studies are recommended to be conducted in other areas. Health authorities should take measures to limit stray dogs and vaccinate dogs. Broadcasting educational programs through mass media about the complications of animal bites and how to prevent is recommended. Further studies are recommended in other regions.

## Data Availability

Data is available by contacting the corresponding author.

## Abbreviations

AAPC: Average annual percent change
HRIG: Human Rabies Immunoglobulin
KUMS: Kermanshah University of Medical Sciences
